# Effect of Different Ammonia Concentrations on Community Succession of Ammonia-oxidizing Microorganisms in a Simulated Paddy Soil Column

**DOI:** 10.1371/journal.pone.0044122

**Published:** 2012-08-31

**Authors:** Hu Baolan, Liu Shuai, Shen Lidong, Zheng Ping, Xu Xiangyang, Lou Liping

**Affiliations:** Department of Environmental Engineering, Zhejiang University, Hangzhou, Zhejiang, China; University of Vienna, Austria

## Abstract

Ammonia oxidation is performed by both ammonia-oxidizing bacteria (AOB) and ammonia-oxidizing archaea (AOA). To explore the effect of ammonia concentration on the population dynamic changes of ammonia-oxidizing microorganisms, we examined changes in the abundance and community composition of AOA and AOB in different layers. Most of the archaeal *amoA* sequences were *Nitrosotalea*-related and the proportion that *Nitrosotalea* cluster occupied decreased in the surface layer and increased in the deep layer during the cultivation process. *Nitrosopumilus*-related sequences were only detected in the deep layer in the first stage and disappeared later. Both phylogenetic and quantitative analysis showed that there were increased *Nitrosomonas*-related sequences appeared in the surface layer where the ammonia concentration was the highest. Both AOA and AOB OTU numbers in different layers decreased under selective pressure and then recovered. The potential nitrification rates were 25.06 µg·N·L^−1^·g^−1^ dry soil·h^−1^ in the mid layer which was higher than the other two layers. In general, obvious population dynamic changes were found for both AOA and AOB under the selective pressure of exogenous ammonia and the changes were different in three layers of the soil column.

## Introduction

Nitrification is critical in the global nitrogen cycle and is a unique pathway linking reduced inorganic nitrogen with oxidised inorganic nitrogen in nature [1]. Ammonia oxidation is the first and rate-limiting step of nitrification. It was assumed that all ammonia oxidisers were bacteria until ammonia-oxidizing archaea were found. The findings of Venter *et al*. [2] and Treusch *et al*. [3] indicated the posibility of archaea involving in ammonia oxidation and the exist of ammonia-oxidation archaea was finally certified by the enrichments and pure cultures of several strains of AOA [4]–[10]. Recent research has demonstrated that ammonia-oxidizing archaea may form a separate and deep-branching phylum, the Thaumarchaeota [11]–[13].

Molecular biological research based on the functional gene *amoA* and the 16SrRNA gene has revealed a widespread distribution of AOA [14]–[17]. Quantitative PCR analyses found that AOA normally outnumbered their substrate competitors, such as AOB, in both soil [17] and water [18], but the relative contributions of AOA and AOB in the soil nitrogen cycle remained undetermined. Some researchers believed that AOB played a more important role than AOA in soil nitrification. Jia and Conrad [19] found that changes in potential nitrification rates were only correlated with the number of AOB *amoA* genes when they added substrate (NH_3_) or inhibitor (C_2_H_2_) of ammonia oxidation to the soil. Subsequently, Di *et al*. [20] discovered the same phenomenon in New Zealand grassland. In contrast, other researchers regarded AOA as the main drivers of ammonia oxidation in soil. Gubry-Rangin *et al*. [21] reported that the potential nitrification rates were positively correlated with the number and transcriptional activity of AOA *amoA* genes. Zhang *et al*. [22] confirmed the dominant role of AOA in soil nitrification by means of stable isotope probe and the quantitative analysis of archaral *amoA* genes. Offre *et al*. [23] also demonstrated that the growth of only archaeal but not bacterial ammonia oxidizers occurred in microcosms with active nitrification. Differences in cell size, specific cell activity and other physiological characteristics may explain the different contributions of AOA and AOB to soil nitrification. In addition, AOA and AOB may compete mutually or exhibit functional redundancy under some conditions, whereas under other conditions the fundamental physiological differences between these organisms may lead to niche separation of AOA and AOB [24]. Therefore, environmental factors are important in determining the different nitrification activities and relative contributions of AOA and AOB.

Among all environmental factors, ammonia is the substrate of ammonia oxidation for which concentrations will directly affect nitrification activity. It was found that AOA were able to grow well and the growth of AOA was coupled with soil nitrification when the concentration of ammonia was relatively low or the supply of ammonia was through the mineralization of organic matter; however, AOB were more competitive in soil nitrification and the number of AOB *amoA* gene copies was greater than that of AOA when the concentration of ammonia was higher [19], [20], [23]–[26]. Adaptation to long-term energy stress is believed to be a crucial factor that distinguishes AOA from AOB [27]. Different specific affinities for substrate between AOA and AOB may explain their different growth patterns under low or high ammonia concentrations. *N. maritimus* exhibited a high affinity for ammonia and was able to grow and convert ammonia at an extremely low ammonia concentration (0.14 µg/L), while the ammonia-oxidizing activity was completely inhibited when the ammonia concentration reached 28 mg/L. The affinity of *N. maritimus* for ammonia can be more than 1,000-fold greater than that of *N. europaea* [28]. The affinity for ammonia of *N. viennensis* fell in between *N. maritimus* and *N. europaea*, and their growth was totally inhibited at ammonia concentration of 280 mg/L [7]. Other than ammonia concentration, other environmental traits such as pH [29], oxygen concentration [18] and organic carbon [30] could affect the abundance and diversity of ammonia-oxidizing microorganisms and consequently lead to niche separation of AOA and AOB.

Paddy soils are an important component of agricultural ecosystems in Asia, and these soils represent one of the major cultivated soil types in China. China has a long history of rice production, and the total area of the paddy soils in the country is 46 M ha [31]. Thus, research on nitrification in paddy soils is of great significance for improving our understanding of nitrogen fluxes in agricultural and soil ecosystems. The abundance and community structure of ammonia-oxidizing microorganisms in paddy soils have been described [32], but the mechanism underlying the response of these microorganisms to ammonia stress is unclear. Therefore, the main objective of this study was to verify the significant role that ammonia concentrations play in the population shifts of AOA and AOB in paddy soils.

## Materials and Methods

### Ethics Statement

No specific permits were required for the described field studies.

### Simulated Soil Column and Sampling

The flooded paddy soil used in this study was collected from paddy land (30°26'N, 120°19'E) in Zhejiang Province, China. The upper 50 cm of paddy soil was collected and filled into the soil column. The soil column was divided into three layers due to the definition and classification of soil layer in pedology and the setting of sampling ports, the surface layer (2–5 cm), the mid layer (17–20 cm) and the deep layer (47–50 cm), to investigate the differences in ammonia-oxidizing microorganisms at different depths.

**Figure 1:**
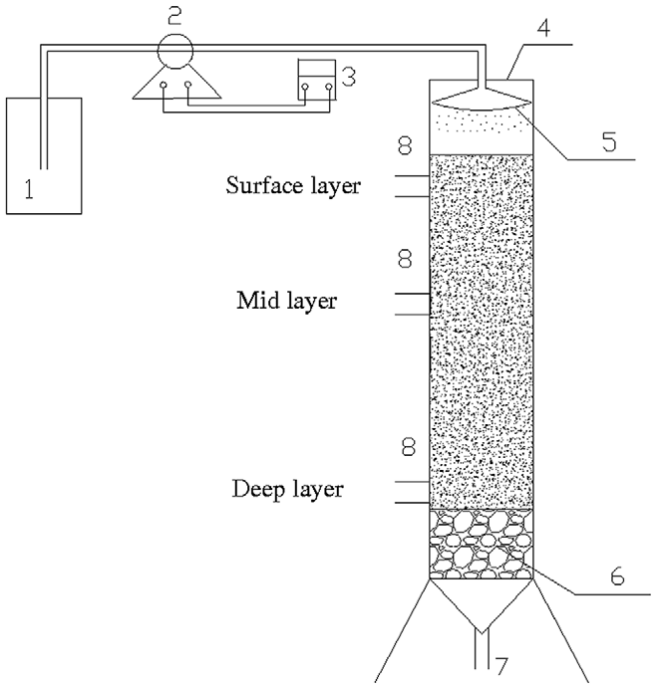
The simulated paddy soil column used in this study. (1) inlet pail, (2) peristaltic pump, (3) timer, (4) main body of soil column, (5) sprayer, (6) beaumontage, (7) outlet pipe, (8) sample connection.

The simulated soil column (Fig. 1) was made of plexiglass and had a height of 70.0 cm and a diameter of 12.5 cm.It had a working volume of 2.5 L and a total volume of 3.0 L. Spherical fillings placed at the bottom of the column acted as a filter to avoid loss of biomass. Paddy soil in agricultural ecosystem were usually irrigated with water from river or rainfall, so natural river water were used in this study to simulate the in situ environment of paddy soil. Therefore, the experimental medium was prepared with natural river water (NH_4_^+^-N, 0.53±0.1 mg/L; NO_2_-N, 0.1±0.03 mg/L; NO_3_-N, 3.72±0.5 mg/L, filtrated by filter) adding ammonia, with final ammonia concentration of 42 mg/L. medium was introduced at the top of the column by spraying (5L per day), and then permeated to the deeper layer. Due to the consumption of microorganisms and adsorption by the soil, an ammonia concentration gradient formed in the soil column from the surface layer to the deep layer (surface layer, 143 µg NH_4_^+^-N g^−1^ dry soil; mid layer, 61.9 µg NH_4_^+^-N g^−1^ dry soil; deep layer, 39.9 µg NH_4_^+^-N g^−1^ dry soil). The soil column was placed in thermostatic chamber and the temperature was 30±1°C. From the top layer to the deep layer, pH of the soil were 7.24±0.12, 7.41±0.1, 7.09±0.15 and the humidity were 38.7±1%, 36.5±0.8% and 34.4±1.5%, respectively. The oxygen concentration in the top soil of the column was 2.48 mg/L.

Soil sampling was performed every six months, constituting the first stage, the second stage, the third stage and the last stage of the cultivation process. For each layer, triplicate samples were collected in each sampling port shown in Figure1 (from the point close to the sampling connection to the point off the sampling connection) using miniature soil sampler and mixed together. The samples were used to measure ammonium, nitrite and nitrate and a small subsample was stored at −80°C for DNA extraction.

### Potential Nitrification Rates

The potential nitrification rates in the soil layers were determined via batch tests using 150 mL conical flasks containing 80 mL of liquid. The medium for determining the rates contained 3.5 mg/L NH_4_^+^-N. Medium with sterilized soil was incubated as a control. Tests were performed in triplicate for 8 hours at 30°C in the dark, and the concentrations of NH_4_^+^-N, NO_2_^–^N and NO_3_^–^N were periodically monitored per 1.5 hours during incubation. Potential nitrification rates were characterized on the basis of the consumption of NH_4_^+^-N. In consideration of some ammonia-oxidizing microorganisms which could accumulate substrates intracellularly [33], the results of potential nitrification rates we obtained may be to some extent higher than the real situation.

### DNA Extraction and PCR Amplification

DNA was extracted using the Power Soil DNA Kit (Mo Bio Laboratories, California, USA) following the manufacturer’s instructions. The extracted DNA was examined in a 1.0% agarose gel by electrophoresis.

Primers sets Arch-amoAF, Arch-amoAR [34] and amoA-1F, amoA-2R [35] were used for the amplification of archaeal and bacterial *amoA* genes, respectively. The PCR thermal cycling programs employed were described previously [36]. The amplified products were examined in a 1.0% agarose gel by electrophoresis.

### Cloning and Sequencing

The obtained PCR products were cloned using the pMD19-T vector (TaKaRa, Bio Inc., Shiga, Japan) according to the manufacturer’s instructions. Plasmid DNA was isolated using the Gene JET™ Plasmid Miniprep kit (Fermentas Life Sciences, Burlington, Canada) and was digested with 5 U of *Eco*RI enzyme in *Eco*RI buffer for 1.5 h at 37°C. The digestion products were examined for an insert of the expected size using agarose (1.0%) gel electrophoresis. At least 20 positive clones from each library were randomly selected for sequencing using an ABI3100 automated sequencer (Applied Biosystems, California, USA).

### Phylogenetic Analyses

All of the *amoA* gene sequences obtained were imported into MEGA4.1 to construct alignment files in combination with the most similar environmental sequences and sequences of known strains of AOA and AOB. Phylogenetic trees were constructed using the neighbour-joining method. Bootstrap analysis with 1000 replicates was applied to estimate the confidence values for the tree nodes.

### Statistical Analyses

Operational taxonomic unit (OTUs) was used to determine the archaeal and bacterial *amoA* gene diversity in different soil samples. For both AOA and AOB, researchers found that 1%–3% 16S rRNA distance equate to a 15% *amoA* gene distance [37], [38]. 85% sequence identity at the *amoA* gene level could be regarded as an approximate threshold below which ammonia-oxidizing microorganisms can be assigned to different species. Hence, OTUs were defined by 15% differences for both archaeal *amoA* gene and bacterial *amoA* gene in nucleotide sequences. OTU numbers were determined by the farthest neighbour algorithm in the DOTUR program [39]. The coverage of the clone libraries was calculated as *C*  =  [1 - (*n*_1_ */N*)] ×100, where *n*_1_ is the number of unique OTUs and *N* is the total number of clones in a library.

### Real-time Quantitative PCR (qPCR)

The primer used to quantify the copy numbers of *amoA* genes of AOA and AOB were the same with those used in the clone library construction. Primer pair P128r, P365r designed by Dang [40] was used to quantify the copy numbers of *Nitrosomonas amoA* gene. qPCR was performed using an iCycler iQ5 thermocycler and a real-time detection system (Bio-Rad, California, USA) according to the method described by Francis *et al.* [34], Mc Tavish *et al.* [41] and Dang [40]. Quantification standards consisted of 10^2^–10^8^, 10^1^–10^7^, 10^2^–10^8^ copy number dilution series of plasmid containing inserted *amoA* genes of AOA, AOB and *Nitrosomonas*, respectively. R^2^ and the amplification efficiencies of the AOA, AOB and *Nitrosomonas* standard curves were 0.996, 0.998 and 0.996 (as shown in Figure S3, S4 and S5) and 96%, 89% and 95%, respectively.

### Analytical Methods

Measurements of NO_3_^–^N, NO_2_^–^N and NH_4_^+^-N in the experimental medium were performed according to standard methods [42]. The total solid (TS) content was measured by the weighing method after drying at 105°C. The inorganic nitrogen contents in the soils were determined colorimetrically via flow injection analysis of soil-KCl extracts as previously described [43].

### Accession Numbers

Sequences were deposited in GenBank under accession numbers JQ320498 to JQ320554.

## Results

### Diversity and Phylogenetic Analysis of AOA and AOB

**Figure 2:**
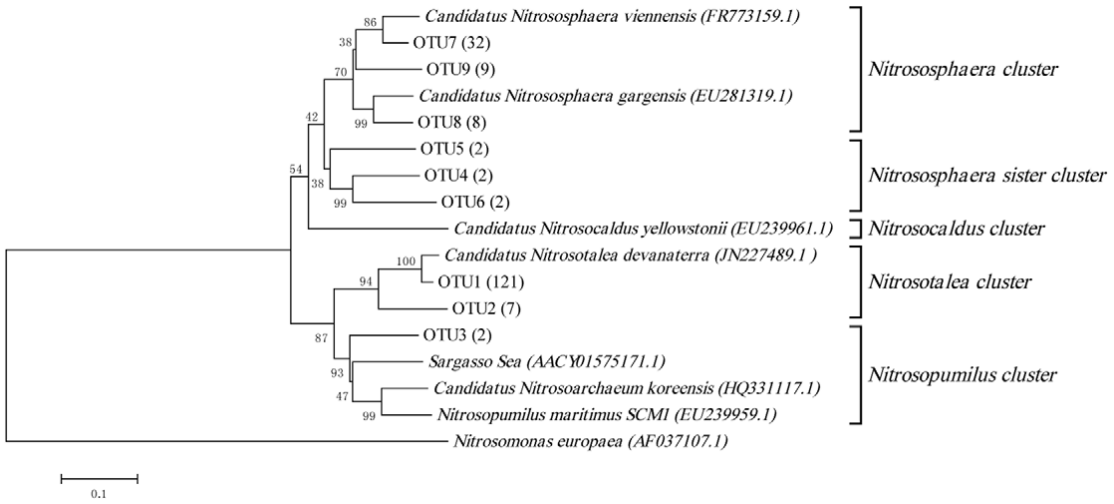
Neighbour-joining phylogenetic tree showing the relationships of the 185 sequences of archaeal *amoA* genes detected in the clone libraries constructed from samples from the soil column with their most similar sequences and sequences of known AOA. The numbers at the nodes are percentages that indicate the levels of bootstrap support from 1,000 replicates. The scale bar represents 0.1 nucleic acid substitutions per nucleotide position.

185 derived archaeal *amoA* genes belonged to *Nitrososphaera* cluster, *Nitrososphaera* sister cluster, *Nitrosotalea* cluster and *Nitrosopumilus* cluster according to a new nomenclature system for archaeal *amoA* genes reported by Pester [37] (Figure 2), but no *Nitrosocaldus*-related sequences was detected. The sequences affiliated with *Nitrosotalea* cluster were the most and accounted for 69.2% of all the archaeal *amoA* genes obtained. Only two sequences were detected belonging to *Nitrosopumilus* cluster and the rest sequences belonged to *Nitrososphaera* cluster and *Nitrososphaera* sister cluster. A total of 9 OTUs were obtained using the DUTOR program based on a 15% *amoA* gene sequence distance cutoff [37] for all 185 AOA *amoA* sequences detected in this study. *Nitrosopumilus* cluster contained only one OTU and the other three clusters contained at least two OTUs. Sequences belonging to OTU1 were found in all three soil layers during the entire cultivation process, totalling 121 sequences, which accounted for 65.4% of the detected AOA *amoA* gene sequences.

**Figure 3:**
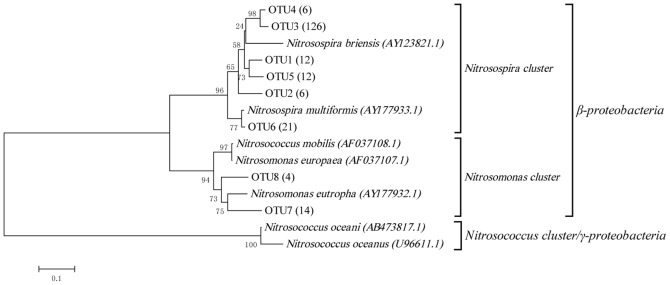
Neighbour-joining phylogenetic tree showing the relationships of the 201 sequences of bacterial *amoA* genes detected in the clone libraries constructed from samples from the soil column with their most similar sequences and the sequences of known AOB. The numbers at the nodes are percentages that indicate the levels of bootstrap support from 1000 replicates. The scale bar represents 0.05 nucleic acid substitutions per nucleotide position.

As to AOB, all 201 sequences obtained for the AOB *amoA* gene in the clone libraries belonged to β-proteobacteria, as shown in Figure 3. Among these sequences, 91.0% were grouped into the *Nitrosospira* genus, and the remainders were grouped into *Nitrosomonas*. A total of 8 OTUs were obtained for the bacterial *amoA* genes using the DUTOR program based on a 15% *amoA* gene sequence distance cutoff [38]. *Nitrosomonas* cluster contained 2 OTUs, and the remainders were grouped into *Nitrosospira* cluster.

### Community Succession of AOA and AOB

**Figure 4:**
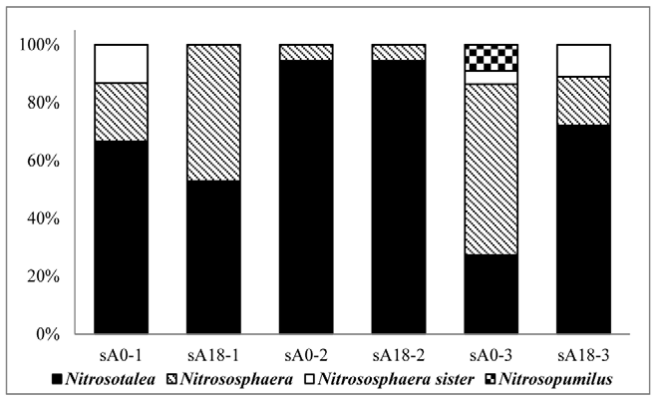
Population shifts of AOA in different layers. (sA0-1 represents the first stage in the surface layer; sA0-2, the first stage in the mid layer; sA0-3, the first stage in the deep layer; sA18-1, the last stage in the surface layer; sA18-2, the last stage in the mid layer; and sA18-3, the last stage in the deep layer).

The community structure of the AOA and AOB in the soil column changed during the cultivation process and was closely related to soil depth. An obvious population shift was found for AOA in different layers when the proportions that each cluster occupied in the first stage and the last stage were compared. In the surface layer, the proportion of *Nitrosotalea* cluster decreased from 66.7% to 52.9% (Figure 4). Accordingly, an increase from 20% to 47.1% was found for *Nitrososphaera* cluster. The sequences affiliated with *Nitrososphaera* sister cluster disappeared during the 18-month cultivation process. Interestingly, in the mid layer, we found that cluster *Nitrosotalea*-related sequences represented more than 94.4% of the sequences detected, and this proportion did not change during the cultivation process. Accordingly, the proportion of *Nitrososphaera*-related sequences was less than 5.6% throughout the cultivation process. In the deep layer, the observed changes were dramatic. In the first stage, the diversity of the population was high and all the four AOA *amoA* clusters were found, the *Nitrosopumilus*-related sequences were only detected in the first stage of the deep layer and represented 9.1% of the sequences detected. However, 18 months later, the sequences belonging to *Nitrosopumilus* disappeared and the proportion that *Nitrosotalea* cluster occupied increased from 27.3% to 72.2%. In the 18-month cultivation process, the number of OTUs in all three layers decreased first and then increased. The number of OTUs in the deep layer was greater than in the other two layers in the first stage (Figure S1). In the second stage, the numbers of OTUs in all three layers were reduced dramatically. In the surface layer, the number of OTUs dropped from 4 to 1, while it decreased from 2 to 1 and from 7 to 3 in the mid and deep layer, respectively. The only difference was that the number of OTUs in the surface and mid layers recovered more rapidly than was observed for the deep layer. The number of OTUs in the surface and mid layers began to increase in the third stage; however, the number of OTUs in the deep layer did not increase until the last stage.

**Figure 5:**
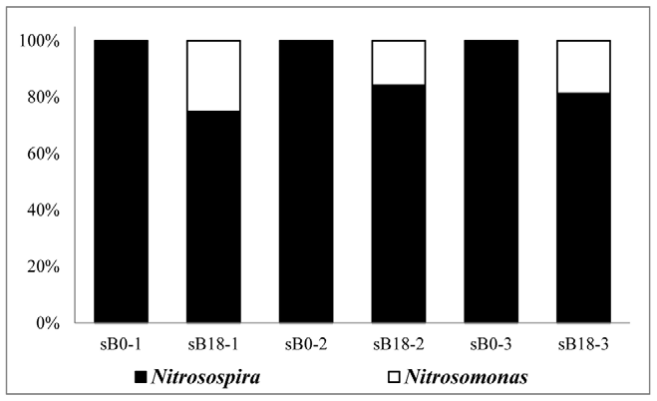
Population shifts of AOB in different layers. (sB0-1 represents the first stage in the surface layer; sB0-2, the first stage in the mid layer; sB0-3, the first stage in the deep layer; sB18-1, the last stage in the surface layer; sB18-2, the last stage in the mid layer; and sB18-3, the last stage in the deep layer).

With respect to AOB, increasing sequences belonging to *Nitrosomonas* were detected in all three layers in the soil column, and the proportions that *Nitrosomonas*-related sequences occupied were 25%, 15.8% and 18.8% from the surface layer to the deep layer (Figure 5). The numbers of AOB OTUs varied in a similar way as was observed for the AOA OTUs. The AOB OTU numbers in the mid and deep layers decreased in the first three stages and increased in the last stage. However, in the surface layer, the number of OTUs remained stable at three after the third stage (Figure S2).

### Real-time Quantitative PCR (qPCR)

**Figure 6:**
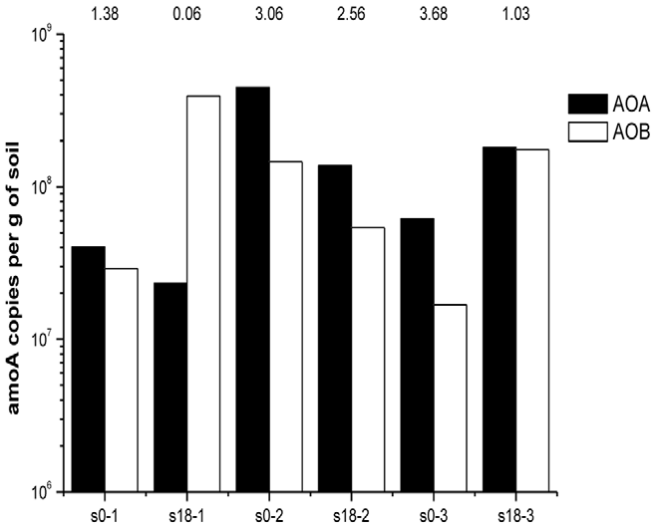
Archaeal and bacterial *amoA* gene copy numbers in different layers. (s0-1 represents the first stage in the surface layer; s0-2, the first stage in the mid layer; s0-3, the first stage in the deep layer; s18-1, the last stage in the surface layer; s18-2, the last stage in the mid layer; and s18-3, the last stage in the deep layer).

**Figure 7:**
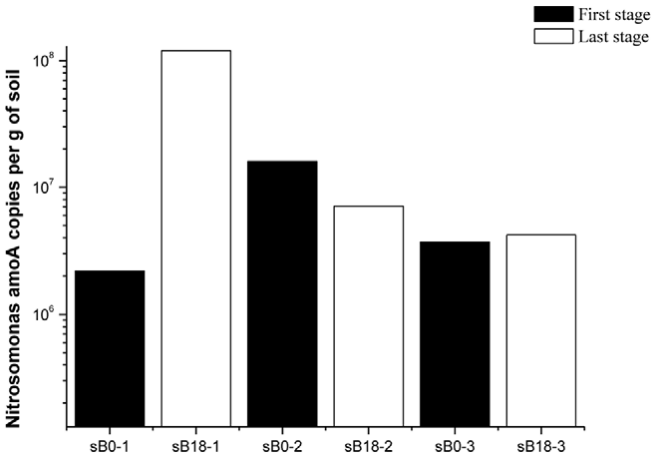
Nitrosomonas amoA gene copy numbers in different layers. (sB0-1 represents the first stage in the surface layer; sB0-2, the first stage in the mid layer; sB0-3, the first stage in the deep layer; sB18-1, the last stage in the surface layer; sB18-2, the last stage in the mid layer; and sB18-3, the last stage in the deep layer).

The AOA and AOB *amoA* genes in the three soil layers were quantified every 6 months. AOA were more abundant than AOB in almost all soil samples, with the exception of the sample from the surface layer in the last stage, in which the AOB/AOA ratio was 16.95. The ratios of AOA/AOB ranged from 1.03–12.96 in the other 11 soil samples (Figure 6). The copy number of *Nitrosomonas amoA* genes increased substantially in the surface layer during the cultivation process, from 2.2×10^6^ to 1.2×10^8^. As for the other two layers, the change of was not obvious (Figure 7).

### Nitrification Activity

Nitrification activity was tested after the 18-month cultivation period. Samples were collected from the three different layers of the soil column. Different nitrification activities were observed for the three layers of the soil column. The maximum specific nitrification activity in the mid layer was 25.06 µg·N·L^−1^·g^−1^·h^−1^, while those in the surface and deep layers were 16.13 µg·N·L^−1^·g^−1^·h^−1^ and 9.97 µg·N·L^−1^·g^−1^·h^−1^, respectively. Therefore, ammonia-oxidizing microorganisms in the mid layer exhibited the highest nitrification activity among the three layers of the soil column.

## Discussion

### Community Succession of AOA and AOB

It was found that 69.2% sequences derived in our soil column were related to *Nitrosotalea devanaterra*-the only ammonia-oxidizing archaea from acid soil [6]. This was in accordance with the high-throughput sequencing results reported by Gubry-Rangina [44], which demonstrated that *Nitrosotalea devanaterra*-related sequences may be predominant in some agricultural soils. pH in our soil column were neutral or slightly alkaline and was not in accordance with the acid environment where *Nitrosotalea devanaterra* was found. The contradictory could be explained by that there may be existed AOA belonging to *Nitrosotalea devanaterra* cluster which could survive and oxidize ammonia to nitrite in neutral or slightly alkaline environment. The community composition of the AOA in the different layers of the soil column underwent great changes during the cultivation process. In the surface layer, the sequences belonging to *Nitrososphaera* sister cluster disappeared after 18-month cultivation. Since there was no isolated or enrichment strain of this AOA cluster [37], the physiochemical characteristics of *Nitrososphaera* sister cluster was unknown. Combined with the increased *Nitrososphaera*-related sequences in the surface and the decreased *Nitrososphaera* sister-related sequences in the deep layer, we deduced that the AOA affiliated with *Nitrososphaera* sister cluster had relative high affinity for ammonia and prefer to live in the environment with relative low ammonia concentration. *Nitrososphaera viennensis* [7] could grow well in the media containing ammonia concentration as high as 15 mM, indicating a low affinity of this strain of AOA. So, it was reasonable that the proportion *Nitrososphaera*-related sequences occupied increased in the last stage compared with the first stage in the surface layer, since it preferred conditions with relatively higher ammonia concentration. Correspondingly, in the deep layer, the proportion *Nitrososphaera*-related sequences occupied decreased from 59.1% to 16.7%. *Nitrosopumilus maritimus* showed extremely high affinity for ammonia and the ammonia inhibition concentration was as low as 2 mM to 3 mM [5], so it was not strange that the *Nitrosopumilus*-related sequences disappeared in the deep layer after exogenous ammonia was added during the cultivation process. No *Nitrosopumilus*-related sequence was detected in the surface and mid layer during the whole 18 months. *Nitrosotalea*-related sequences were the dominant species in our soil column throughout the cultivation process. In the surface layer, the proportion that the *Nitrosotalea*-related sequences occupied decreased from 66.7% to 52.9%; on the contrary, the proportion in the deep layer increased from 27.3% to 72.2%. It was reported that the acid AOA could grow well with the total NH_3_& NH_4_^+^ concentration ranged from 10 µM to 10 mM (equivalent to 0.18 nM to 0.18 µM of NH_3_) at pH 4.5 and no growth was detected at 50 mM and 100 mM (equivalent to 9 µM to 18 µM NH_3_, pK_a_ for NH_3_: NH_4_^+^, 9.25) [6]. The available NH_3_ concentration in the medium was 44µM (pH, 7.24; 30°C), which was much higher than the ammonia inhibition concentration (9 µM) of *Nitrosotalea devanaterra*. So, the sequences affiliated with *Nitrosotalea* cluster decreased in the surface layer where the ammonia concentration was relatively higher during 18 months cultivation process.

In our simulated soil column, we found that all three layers contained a certain amount of *Nitrosomonas*-like sequences after 18 months. In the surface layer the proportion occupied by *Nitrosomonas*-like sequences was 25.0%, which was higher than in the other two layers (mid layer, 15.8%; deep layer, 18.8%). Quantitative analysis also reached the similar conclusion that the copy number of *Nitrosomonas amoA* genes increased from 2.2×10^6^ to 1.2×10^8^ in the surface layer, and the copy numbers were much higher than that in the other two layers. This could be explained by differences in physiological characteristics between *Nitrosomonas* and *Nitrosospira*. Schramm *et al*. [45], [46] have reported that the growth of *Nitrosomonas* species followed an r-strategy. The *Nitrosomonas* genus had a low ammonia affinity and was adaptable to conditions where the concentration of ammonia was high. In contrast, the growth of *Nitrosospira* species followed a k-strategy. The *Nitrosospira* genus had a high ammonia affinity and was adaptable to conditions where the concentration of ammonia was low. The appearance of and increases in *Nitrosomonas*-like sequences were reflection of the competitive advantage of *Nitrosomonas* species under higher ammonia concentrations when exogenous ammonia was added. The obtained *Nitrosomonas*-like sequences were primarily found in the surface layer because of the relatively higher ammonia concentration in this layer compared with the other two layers. Notably, no *Nitrosomonas*-related sequences was found in the first stage through construction of AOB clone libraries, but we indeed detected *Nitrosomonas amoA* gene using qPCR, this may be due to the different specificities of the primers used in phylogenetic and quantitative analysis.

The richness of AOA and AOB decreased under ammonia stress and then recovered after a period of adaptation, as reflected in the variation in OTU numbers. It seemed that the AOA in the deep layer were less sensitive to exogenous ammonia than those in the other two layers and had a relative longer adaptation period. Another explanation of the OTU numbers variation was the cultivation effect that led to the survival of only a few adapted species which take over the community. For instance, in the deep layer of the first stage, AOA population was constituted with OTU1-3 and OTU6-9, and 6 months later just OTU1, OTU7 and OTU9 were left. In the last stage, the population was composed with 4 OTUs and a new OTU was found even though it had never been derived in the first three stages, so finally the population was taken over by the survival species who could adapt to the selective pressure of the environment.

### Abundance of AOA and AOB and Potential Nitrification Rates (PNR) in Different Layers

The ratio of AOA/AOB in soils was first reported by Leininger in 2006 [17]. He found that the ratio of AOA/AOB was correlated with soil depth in long-term fertilized soils and AOA *amoA* gene copy numbers could be three orders of magnitude greater than AOB *amoA* gene numbers at a depth of 30–40 cm. In the present study, the highest ratio of AOA/AOB was found to be 12.96 in the second stage in the surface layer. The growth and ammonia oxidation activity of *Nitrosopumilus maritimus* were inhibited at an ammonia concentration of 28 mg/L [28] which was lower than the inhibitory concentrations (7000–14000 mg/L) of ammonia found for AOB [47]. Therefore, it was expected that the numbers of bacterial *amoA* gene copies would exceed the archaeal *amoA* gene copy numbers in the surface layer during the last stage, where the ammonia concentration was the highest. The potential nitrification rates (PNR) were highest in the mid layer and were 1.6 times higher than that in the surface layer and 2.5 times higher than that in the deep layer. The lower potential nitrification rates in the deep layer may be the cause of the change in redox potential along with soil depth, as reported previously [48]. The redox potential in soils is determined by the relative concentrations of substances in an oxidized state versus a reduced state, which are influenced by oxygen concentrations. The deep layer exhibited the lowest redox potential, which resulted from the lowest oxygen concentration. The fact that the lowest pH (7.09±0.15) existed in the deep layer compared with the other two layers (surface layer, 7.24±0.12; mid layer, 7.41±0.1) may also play an important role.

### Conclusion

In general, obvious population dynamic changes were found for both AOA and AOB under the selective pressure of exogenous ammonia and the changes were different in three layers of the soil column.

## Supporting Information

Figure S1 **Changes in AOA OTU numbers in different layers during the 18-month cultivation process.**
(TIF)

Figure S2 **Changes in AOB OTU numbers in different layers during the 18-month cultivation process.**
(TIF)

Figure S3 **The standard curve of qPCR for AOA** ***amoA*** **genes.**
(TIF)

Figure S4 **The standard curve of qPCR for AOB** ***amoA*** **genes.**
(TIF)

Figure S5 **The standard curve of qPCR for** ***Nitrosomonas amoA*** **genes.**
(TIF)
