# Supplementary figures and images for: Effect of Different Ammonia Concentrations on Community Succession of Ammonia-oxidizing Microorganisms in a Simulated Paddy Soil Column
Source: PLoS One. 2012 Aug 31;7(8):e44122. doi: 10.1371/journal.pone.0044122 (PMC3432066; doi:10.1371/journal.pone.0044122)

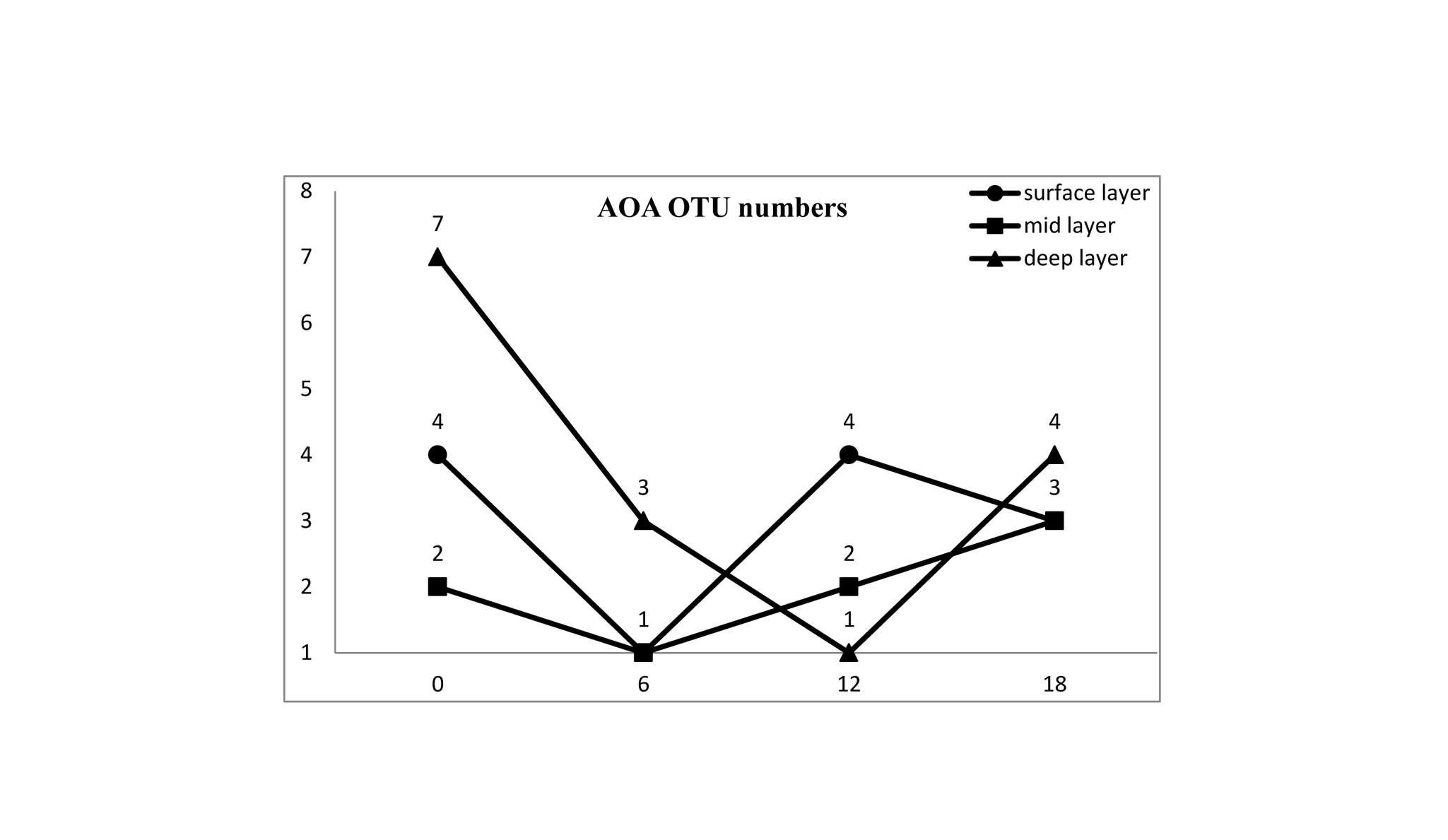

Supplement: Figure S1 — Changes in AOA OTU numbers in different layers during the 18-month cultivation process. (TIF) [file pone.0044122.s001.tif]

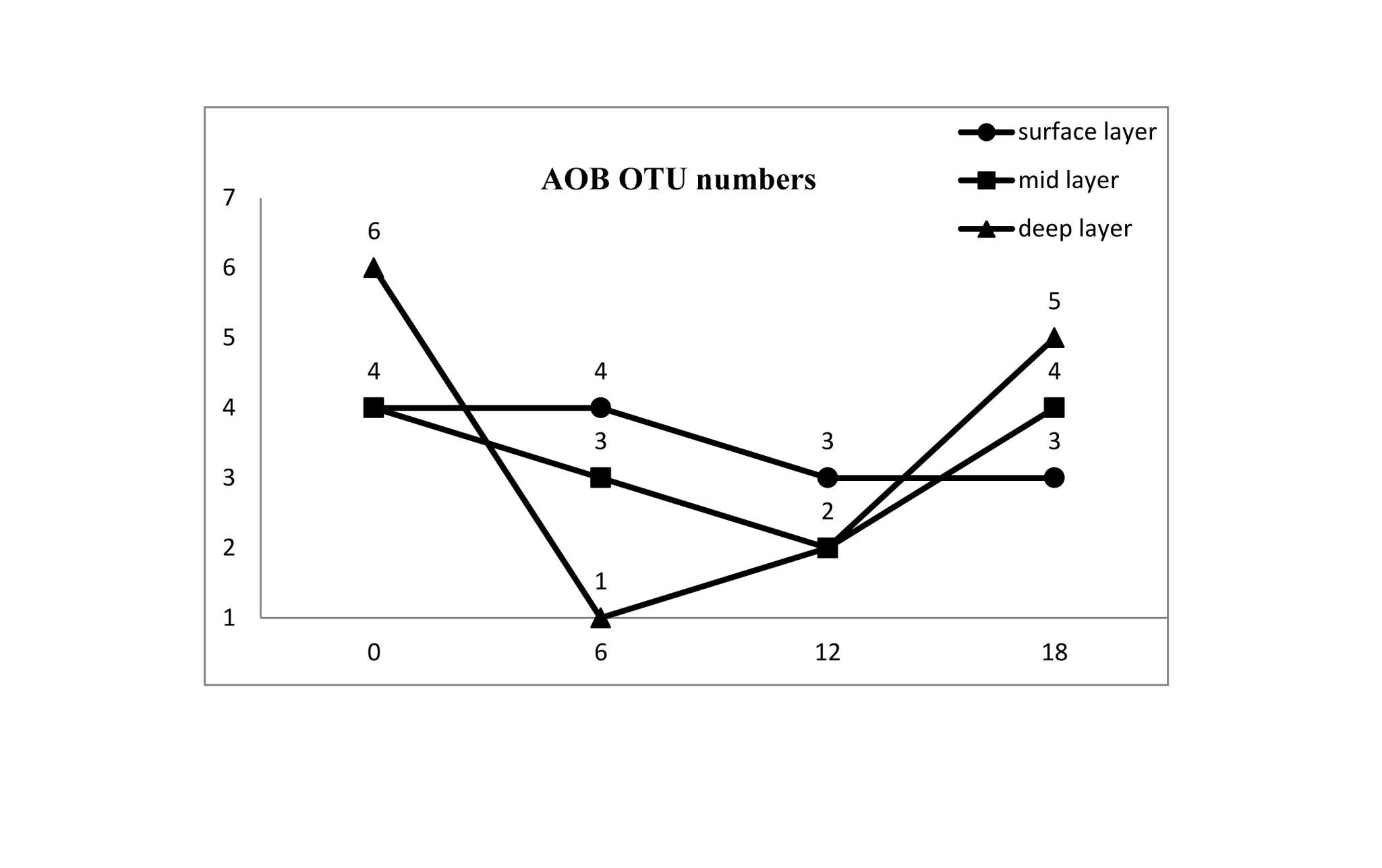

Supplement: Figure S2 — Changes in AOB OTU numbers in different layers during the 18-month cultivation process. (TIF) [file pone.0044122.s002.tif]

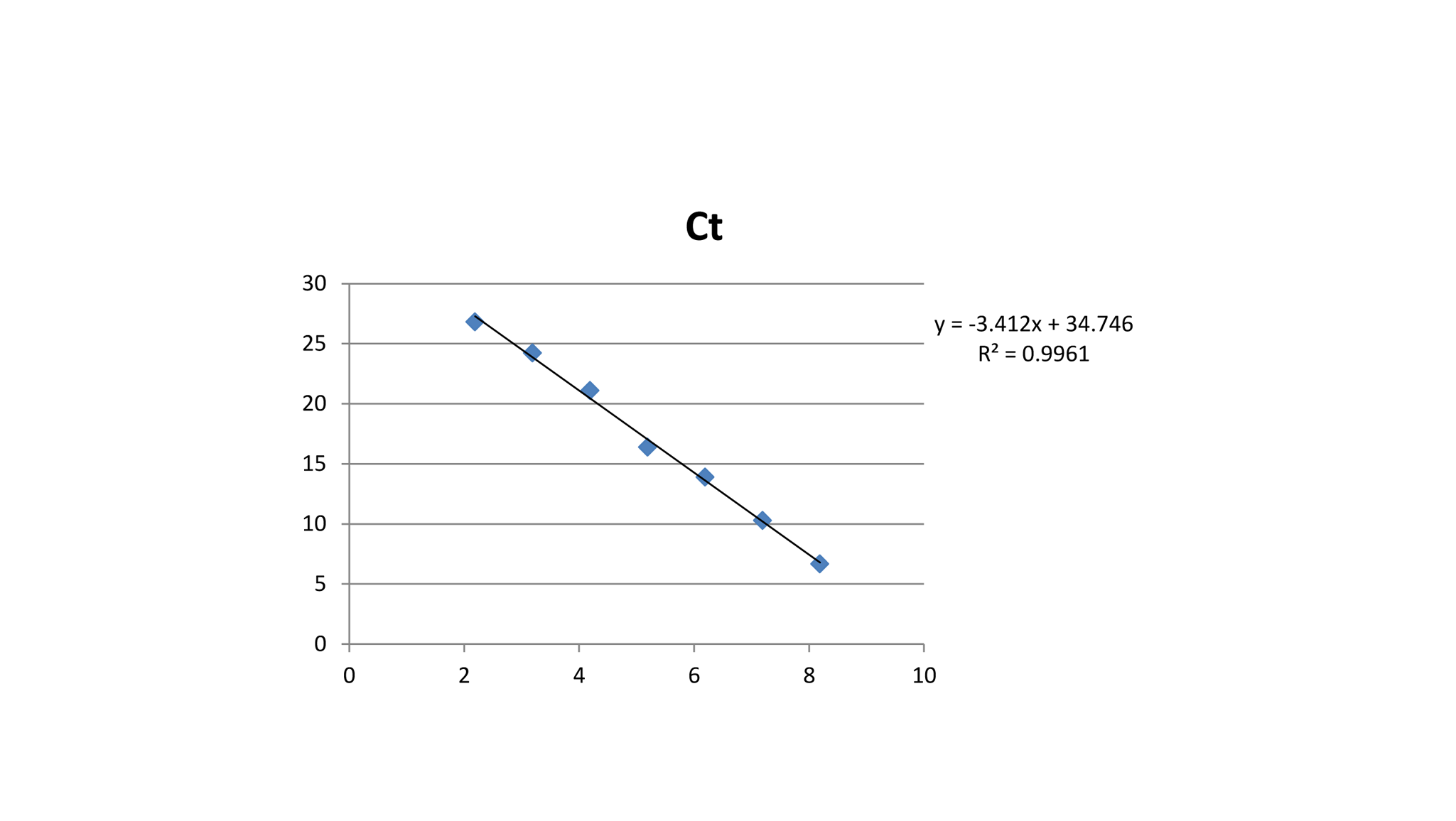

Supplement: Figure S3 — The standard curve of qPCR for AOA amoA genes. (TIF) [file pone.0044122.s003.tif]

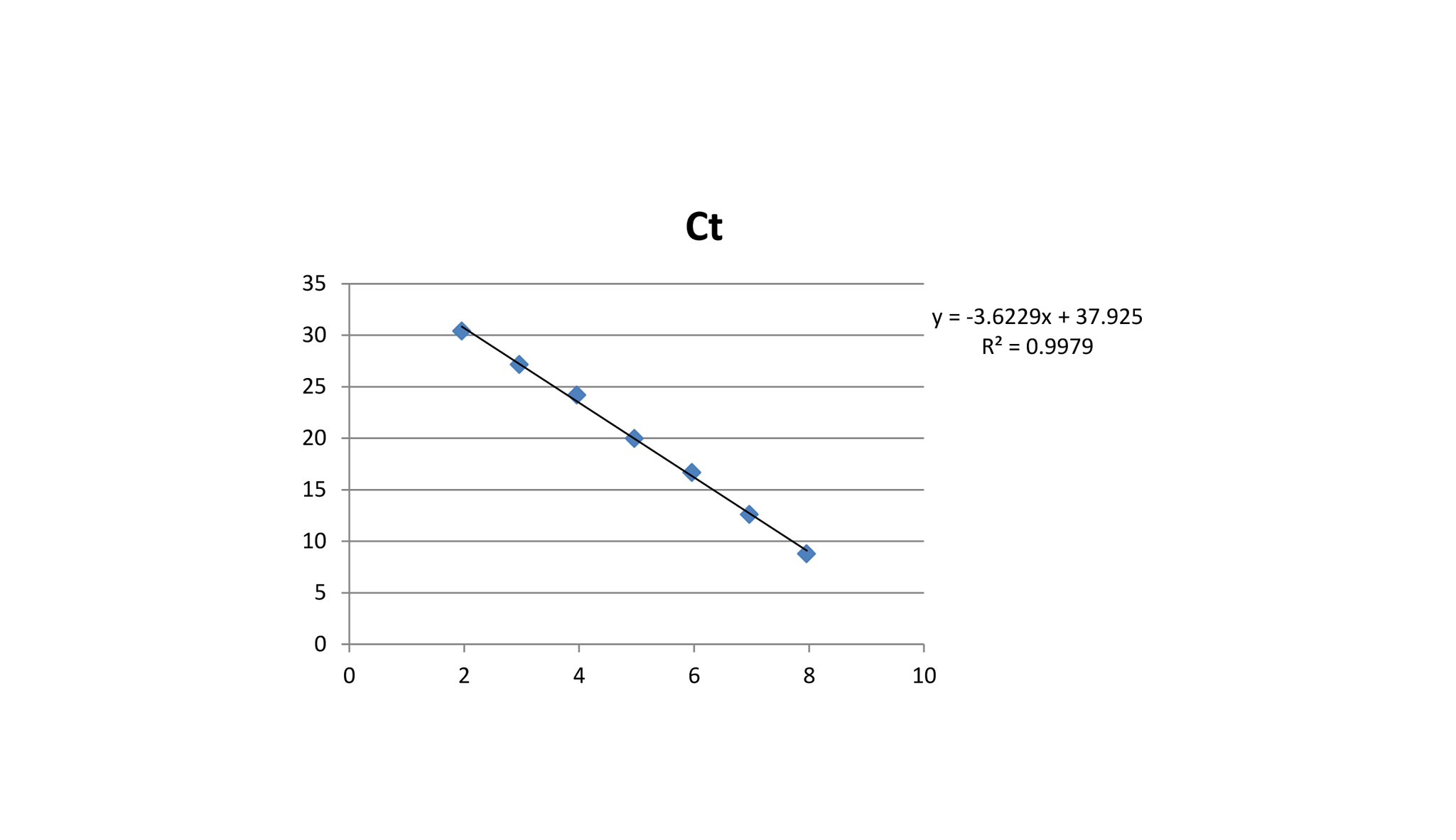

Supplement: Figure S4 — The standard curve of qPCR for AOB amoA genes. (TIF) [file pone.0044122.s004.tif]

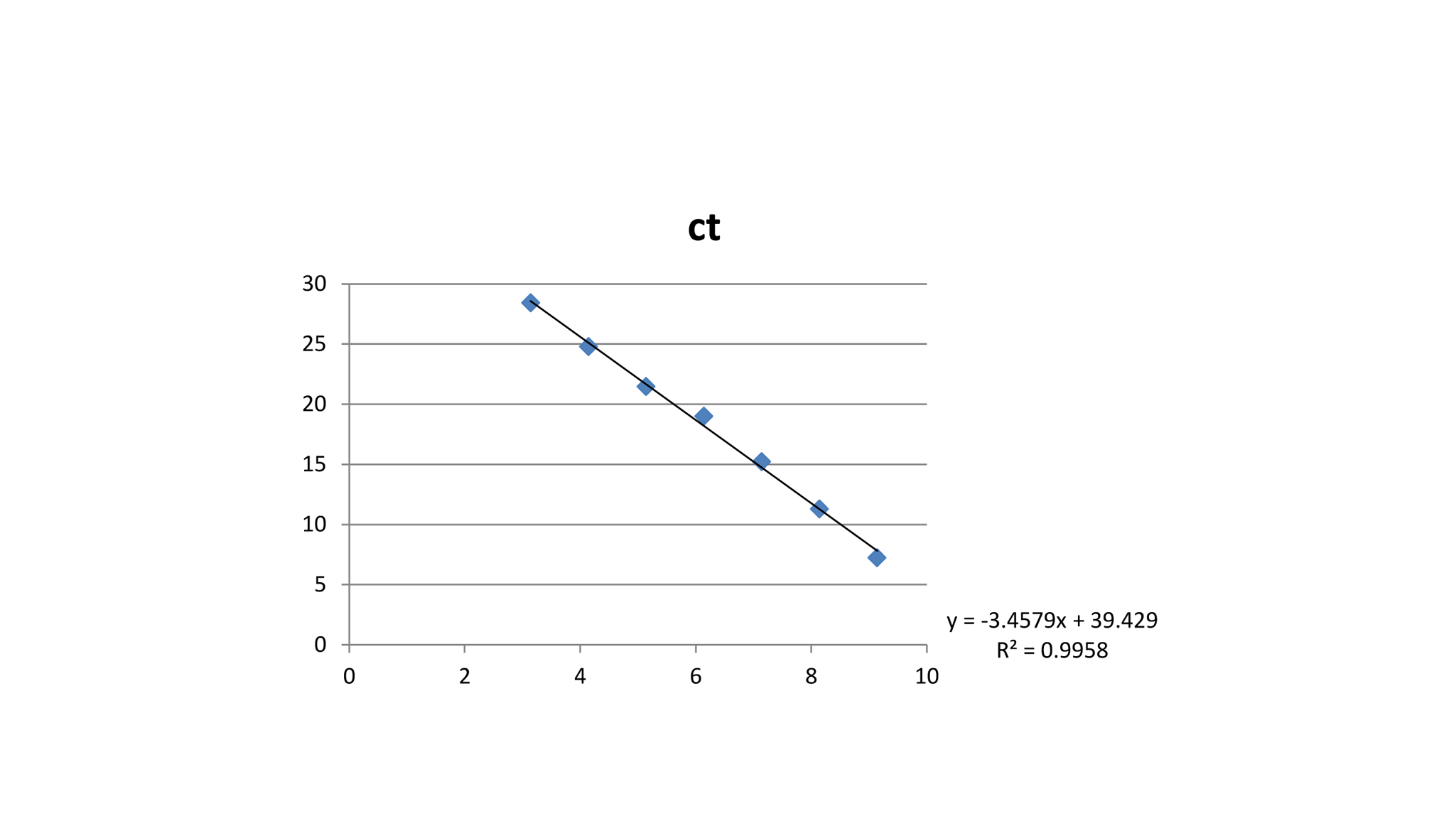

Supplement: Figure S5 — The standard curve of qPCR for Nitrosomonas amoA genes. (TIF) [file pone.0044122.s005.tif]
